# The Influence of C-Ions and X-rays on Human Umbilical Vein Endothelial Cells

**DOI:** 10.3389/fonc.2016.00005

**Published:** 2016-01-20

**Authors:** Alexander Helm, Ryonfa Lee, Marco Durante, Sylvia Ritter

**Affiliations:** ^1^Department of Biophysics, GSI Helmholtz Centre for Heavy Ion Research, Darmstadt, Germany; ^2^Department of Condensed Matter Physics, Technical University of Darmstadt, Darmstadt, Germany

**Keywords:** cardiovascular disease, endothelial cells, high-LET radiation, carbon ions, carbon ion therapy, chromosome 13, micronucleus formation, senescence-associated β-galactosidase

## Abstract

Damage to the endothelium of blood vessels, which may occur during radiotherapy, is discussed as a potential precursor to the development of cardiovascular disease. We thus chose human umbilical vein endothelial cells as a model system to examine the effect of low- and high-linear energy transfer (LET) radiation. Cells were exposed to 250 kV X-rays or carbon ions (C-ions) with the energies of either 9.8 MeV/u (LET = 170 keV/μm) or 91 MeV/u (LET = 28 keV/μm). Subculture of cells was performed regularly up to 46 days (~22 population doublings) post-irradiation. Immediately after exposure, cells were seeded for the colony forming assay. Additionally, at regular intervals, mitochondrial membrane potential (MMP) (JC-1 staining) and cellular senescence (senescence-associated β-galactosidase staining) were assessed. Cytogenetic damage was investigated by the micronucleus assay and the high-resolution multiplex fluorescence *in situ* hybridization (mFISH) technique. Analysis of radiation-induced damage shortly after exposure showed that C-ions are more effective than X-rays with respect to cell inactivation or the induction of cytogenetic damage (micronucleus assay) as observed in other cell systems. For 9.8 and 91 MeV/u C-ions, relative biological effectiveness values of 2.4 and 1.5 were obtained for cell inactivation. At the subsequent time points, the number of micronucleated cells decreased to the control level. Analysis of chromosomal damage by mFISH technique revealed aberrations frequently involving chromosome 13 irrespective of dose or radiation quality. Disruption of the MMP was seen only a few days after exposure to X-rays or C-ions. Cellular senescence was not altered by radiation at any time point investigated. Altogether, our data indicate that shortly after exposure C-ions were more effective in damaging endothelial cells than X-rays. However, late damage to endothelial cells was not found for the applied conditions and endpoints.

## Introduction

An increased risk of cardiovascular disease (CVD), i.e., any disease involving the heart or blood vessels, such as ischemic heart disease, myocardial infarction, or hypertension, is a known consequence of radiotherapy for the treatment of certain types of cancer, such as breast cancer or Hodgkin lymphoma, where the heart is typically part of the radiation field and thus may be exposed to relatively high doses of ionizing radiation (IR) (1, 2). Although modern radiotherapy techniques aim to spare organs at risk such as the heart, coronary arteries may still be affected and thus a risk for cardiovascular damage remains (3, 4). Furthermore, there is growing evidence of an increased risk of CVD at low and moderate doses of IR stemming mainly from atomic bomb survivors and occupationally exposed groups, typically developing with a long latency (5–7). Generally, radiation-induced cell killing of endothelial cells and a subsequent induction of a pro-inflammatory response are considered as the mechanism triggering arteriosclerosis and ischemic heart disease (6, 8, 9). The mechanisms by which low and moderate doses of IR provoke CVD are still poorly understood. However, direct damage to endothelial cells followed by an inflammatory response seems to play also a role at low doses (6, 10).

Radiation-induced damage to the endothelium may simply be a consequence of cell loss due to cell killing, as discussed by Little et al. (6). Yet, also radiation-induced genomic instability, oxidative stress disrupting mitochondrial function, and accelerated cellular senescence have been implicated in the pathogenesis of arteriosclerosis (8, 11–14). So far, most data are available on the effects of low-linear energy transfer (LET) radiation, while only few data on the impact of high-LET radiation exist, yet suggesting a higher risk (10). With an increasing use of high-LET particles such as carbon ions (C-ions) in cancer therapy or radiosurgery (15–17), an assessment of their possible cardiovascular effects is important.

To gain a deeper insight into the effects of high-LET radiation on endothelial cells, we chose human umbilical vein endothelial cells (HUVEC) as a model system. HUVEC have been already used to study the radiation response to both low- and high-LET radiation investigating, e.g., cell survival, apoptosis, gene expression, or angiogenesis [e.g., Ref. (18–20)]. We exposed cells to C-ions with two different energies relevant for cancer therapy, i.e., 9.8 and 91 MeV/u corresponding to LET values of 170 and 28 keV/μm. For comparison, X-ray experiments were performed. The focus was set on doses ≤1.5 Gy. We investigated clonogenic cell survival, apoptosis, and cytogenetic damage expressed as micronuclei formation or chromosomal aberrations, premature senescence, and the integrity of the mitochondrial membrane potential (MMP). Measurements were performed up to 46 days post-irradiation.

## Materials and Methods

### Cell Culture

Human umbilical vein endothelial cells were purchased from PromoCell (Heidelberg, Germany) and cultured according to the manufacturer’s protocol in medium optimized for the cultivation of primary endothelial cells from large blood vessels. Briefly, cells were maintained in basal Endothelial Cell Growth Medium supplemented with Endothelial Cell Growth Kit components. The final supplement concentrations in the medium were 2% fetal calf serum, 0.1 ng/ml epidermal growth factor, 1 μg/ml hydrocortisone, 1 ng/ml basic fibroblast growth factor, and 0.4% endothelial cell growth supplement. Cells were passaged every 4–5 days upon reaching ~80% confluency. For cell detachment, a mixture of 0.05% trypsin and 0.02% EDTA was used and neutralized with trypsin neutralizing solution containing 0.05% trypsin inhibitor in 0.1% BSA and plated at a density of 6.6 × 10^3^ cells/cm^2^ unless otherwise stated. Medium was changed for every 2–3 days, and the cumulative population doubling (CPD) was determined. All cell culture products were purchased from PromoCell.

### Irradiation

Sub-confluent cultures with a CPD level of about 6 (culture age: about 11 days) were exposed to X-rays or C-ions with an initial energy of either 11.4 or 100 MeV/u at GSI Helmholtz Centre for Heavy Ion Research (Darmstadt, Germany). For the exposure to X-rays or high energy C-ions, cells were seeded into 25 cm^2^ culture flasks, whereas for the exposure to low energy C-ions, cells were plated into 35 mm Petri dishes.

X-ray irradiation was performed at a Seifert (Germany) X-ray machine operated at 250 kV and 16 mA with a 1 mm Al + 1 mm Cu filtering. The dose rate was about 1.5 Gy/min. Exposure to 11.4 MeV/u C-ions was done at the linear accelerator UNILAC, as described in detail elsewhere (21, 22). At sample position, the energy was 9.8 MeV/u corresponding to an LET of 170 keV/μm. Irradiation with 100 MeV/u C-ions was performed at the heavy ion synchrotron SIS with the raster scanning technique (23). The resulting energy on target was 91 MeV/u with an LET of 28 keV/μm. For C-ions, the irradiation time was in the range of 0.5–2 min depending on dose and accelerator conditions. All exposures were done at room temperature, and control samples were sham irradiated.

For longer follow-up studies (up to 46 days post-irradiation corresponding to 22 population doublings), we limited the analyses to doses at an isosurvival level of about 50 and 20%, respectively. Cell survival of 50% was expected for 0.75 Gy X-rays, 0.35 Gy 91 MeV/u C-ions, and 0.25 Gy 9.8 MeV/u C-ions, while a survival rate of 20% was estimated for 1.5, 0.75, and 0.5 Gy, respectively. Further details on particle fluences and the number of particle traversals per nucleus are given in Table S1 in Supplementary Material.

### Clonogenic Cell Survival

Cell survival was measured using the standard colony forming assay (24). In brief, directly after exposure cells were trypsinized, counted, and plated in triplicate into 25 or 75 cm^2^ tissue culture flasks. The number of cells seeded was estimated to result in a statistically significant formation of at least 100 colonies. After 12 days of incubation, cells were fixed and stained. Cell clusters consisting of at least 50 cells were counted as a colony.

### Micronuclei

To assess the cytogenetic damage 24 h after radiation exposure, the micronucleus assay was applied as described in Fenech (25) with minor modifications. Briefly, cells were incubated for 4 h following irradiation and subsequently treated with 0.75 μg/ml cytochalasin-B for 20 h. Cells were then washed in PBS, fixed in 8% formaldehyde for 5 min, and stained with DAPI (0.2 μg/ml) for 15 min at room temperature. At least 1000 cells were scored, and the number of binucleated cells containing micronuclei was determined. For follow-up studies, i.e., >24 h, cells were regularly subcultured and at selected time points the spontaneously occurring frequency of cells carrying micronuclei was analyzed by scoring 1000 cells per dose and time point.

### Apoptosis

For analysis at the early time point, cells were fixed in 8% formaldehyde and stained with DAPI as described for the micronuclei samples. Additionally, cells were subcultered and at consecutive time points 5 × 10^4^ cells were seeded in 35 mm tissue culture dishes and incubated for 2 more days until fixation and staining. At least 1000 cells were scored per dose and time point. Apoptotic cells were identified under a fluorescence microscope (400× magnification) by the typical morphological changes of the cell nucleus, such as chromatin condensation or fragmentation (26, 27).

### Senescence-Associated β-Galactosidase

Analysis of cellular senescence-associated β-galactosidase activity (SA-β-gal) was performed using the Senescence Cell Staining kit (Sigma-Aldrich, Germany) according to the manufacturer’s protocol. At several time points after radiation exposure (2 up to 44 days), cells were seeded at a density of 5 × 10^4^ in 35 mm tissue culture dishes. Two days later, cells were fixed and staining. At least 2000 cells were scored by light microscopy (400× magnification), and the fraction of cells exhibiting a blue stain, i.e., SA-β-gal activity, was determined.

### Mitochondrial Membrane Potential

To assess the influence of radiation exposure on the MMP (also referred to as ΔΨ_M_), the cationic, lipophilic dye 5,5′,6,6′-tetra-chloro-1,1′,3,3′-tetraethylbenzimidazolyl-carbocyanine iodide (JC-1) was applied. The dye shifts its fluorescence signal from 525 nm (green) to 595 nm (red) due to a dimerization in the presence of protons thus indicating a functional MMP. For MMP analyses, samples were collected 12, 24, and 48 h after exposure. Measurements at later time points were performed using ~80% confluent cultures. Analysis of the MMP was performed as described previously (28) with modifications. Briefly, cells were harvested and incubated for 10 min in medium containing JC-1 (5 μg/ml) at 37°C. Thereafter, cells were washed twice with PBS analyzed by flow cytometry using a Pas III Particle Analysing System and the software FloMax (both from Partec, Germany). The fraction of predominantly red cells, i.e., cells mainly containing mitochondria with an intact MMP, was determined in at least 1 × 10^4^ cells of each sample. As a positive control, cells were treated with 2 mM 2,4-dinitrophenol 10 min before JC-1 staining, resulting in ~5% of cells with a red fluorescent signal.

### Chromosome Analysis

Chromosome aberrations were analyzed in control cultures at CPD 13 ± 2 and in the progeny of irradiated cells at CPD 22 ± 2. For cytogenetic analyses, cells were seeded into 75 cm^2^ flasks and cultured for 2 days. Then, colcemid (0.1 μg/ml) was added for 3 h to accumulate metaphase cells. Chromosome spreads were prepared according to the standard procedures, e.g., cells were trypsinized, treated with hypotonic solution, fixed, and dropped on wet slides. Slides were stained using multiplex fluorescence *in situ* hybridization (mFISH). For mFISH analysis, slides were hybridized with the 24XCyte mFISH probe kit from MetaSystems (Altlussheim, Germany) following the instructions of the manufacturer. Chromosome spreads were examined using an Olympus BX61 microscope (Olympus, Tokyo, Japan) equipped with six filter sets specific for the applied fluorochromes. Images of the metaphases were captured (100× objective) with a charged coupled device camera, and karyotyping was performed using the ISIS/mFISH software. Both, structural and numerical aberrations were recorded in at least 100 metaphases per dose and time point. Structural aberrations were classified following the mPAINT system, as described in detail elsewhere (29). In the present study, breaks and simple exchanges were detected. Breaks were referred to as terminal deletions, when the centric and acentric part of the same chromosome were present within the cell. Terminal deletions involved either both chromatids at the same location (chromosome-type breaks, csb) or only one chromatid (chromatid-type break, ctb). Additionally, lone truncated chromosomes (T) were found, i.e., the acentric part of chromosome was not visible. Simple exchanges include translocations (complete, incomplete, and one-way forma) and dicentrics.

### Statistics

When applicable, data were expressed as the mean value ± SEM or SD as indicated. For data stemming from one experiment only, Poisson statistics were applied to calculate the error bars as indicated, and statistical analysis was performed using a Fisher’s exact test as indicated. Survival data have been normalized by evaluating the plating efficiency not considering control data (0 Gy) only, but rather by performing a fit of the form (α × *d* + *o*) to the experimental data, where *o* is an offset term, which reflects the plating efficiency, determined from all data points. This procedure is more precise, as all measured data are subject to the same plating efficiency and consequently all data points can be exploited to derive this quantity. Deviations for 0 Gy to full survival arises, as also control measurements are affected by uncertainty. Based on the α-values derived from the linear fitting, a Student’s *t*-test was used for statistical analysis. Curve fitting of the micronuclei formation 24 h after exposure was performed according to

(1) $$Y=p_{1}D\times e^{-p_{2}D}$$

where *Y* is the yield of micronuclei, *D* the dose, and *p*_1_ and *p*_2_ fitting parameters. Statistical analysis was performed based on the parameters derived from the fitting using a Student’s *t*-test. Generally, differences were considered significant if the *p*-value ≤0.01.

## Results

### Radiation Affects Clonogenic Cell Survival and Micronuclei Formation in a Dose- and LET-Dependent Manner 24 h after Exposure

**Figure 1:**
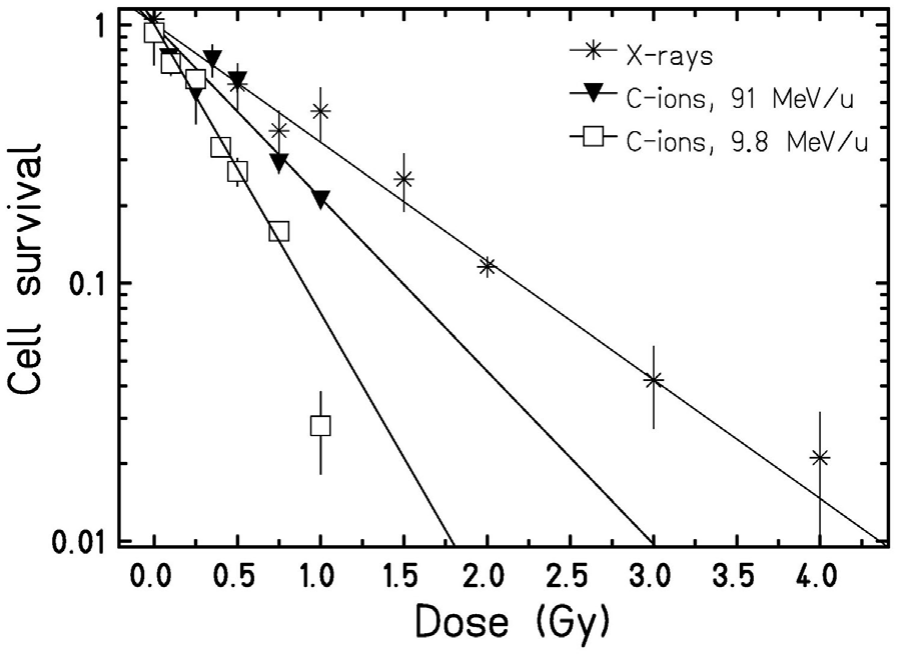
**Clonogenic cell survival of HUVEC**. Cells were plated immediately after exposure to X-rays or C-ions. Data points represent the mean X ± SD from replicates stemming from one (C-ions) or three (X-rays) experiments. Curves were fitted by a linear function. Based on the α-values, clonogenic cell survival was found significantly (*p* < 0.01, Student’s *t*-test) different for the three radiation types.

**Figure 2:**
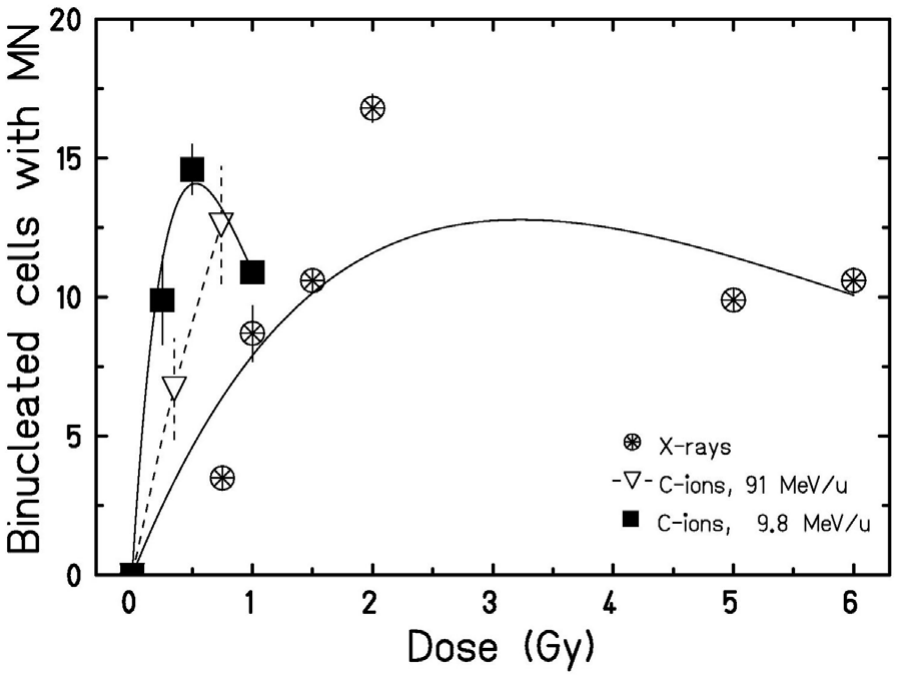
**Micronuclei formation 24 h after exposure**. Following irradiation, cells were incubated with cytochalasin-B, and the amount of binucleated cells containing micronuclei was determined. Data points represent the mean X ± SEM (for data points with *n* = 2) or error was calculated according to Poisson statistics for data points stemming from one experiment. Curves for X-rays and 9.8 MeV/u C-ions were fitted as described. For 91 MeV/u C-ions, lines are drawn to guide the eye. Statistical analysis using a Student’s *t*-test revealed significant differences for 9.8 MeV/u C-ions when compared to X-rays (*p* < 0.01).

**Figure 3:**
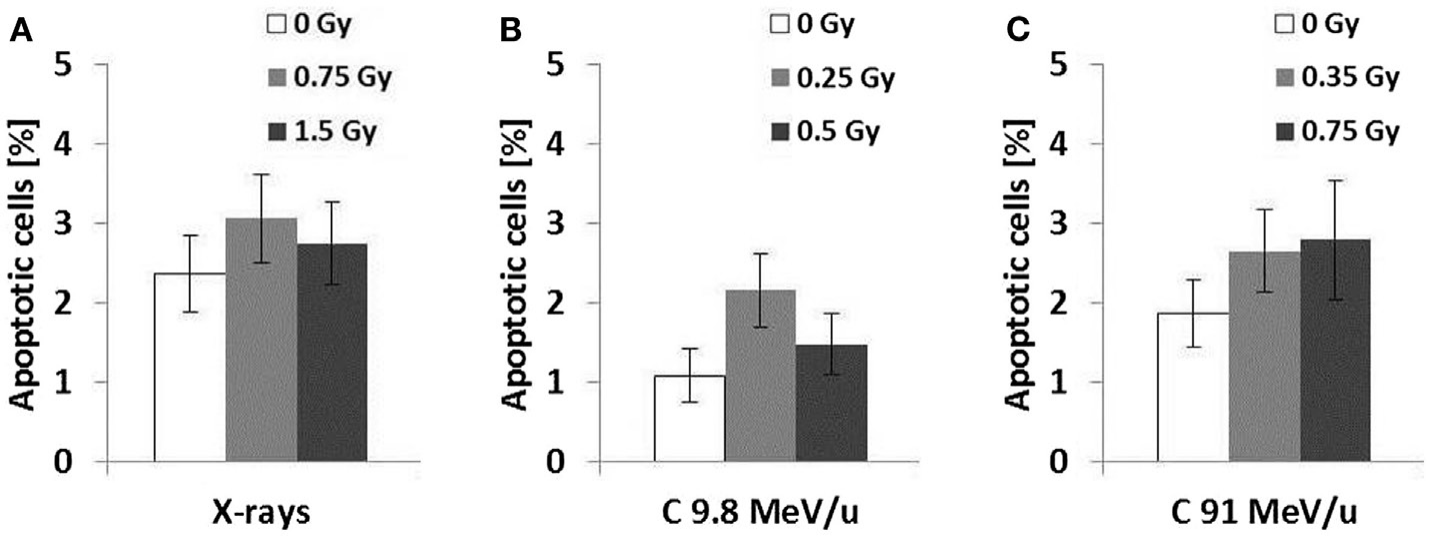
**Apoptosis 48 h after exposure**. Cells were fixed 48 h following radiation exposure to X-rays **(A)**, 9.8 MeV/u C-ions **(B)**, and 91 MeV/u C-ions **(C)**. The fraction of apoptotic cells was determined according to morphological criteria of the nucleus. Error was calculated according to Poisson statistics. Fisher’s exact test revealed no significant differences between all samples (*n* = 1, *p* > 0.01).

To examine the putative radiation effect directly after exposure, a clonogenic cell survival assay was performed (Figure 1). For the three radiation types investigated cell survival decreased with dose and showed a clear LET dependence, i.e., 9.8 MeV/u C-ions with LET = 170 keV/μm were most effective, followed by 91 MeV/u C-ions with LET = 28 keV/μm and X-rays with 2 keV/μm. As all survival curves are linear, the relative biological effectiveness (RBE) does not depend on survival level, resulting in values of 2.4 and 1.5 for 9.8 and 91 MeV/u C-ions, respectively. Next, we measured cytogenetic damage in cells undergoing first division after exposure (24 h after exposure, cytochalasin-B treatment). The analysis showed an LET-dependent formation of micronuclei in binucleated cells (Figure 2). Within the limited dose range examined a saturation in the yield of cells carrying micronuclei was observed for >0.5 Gy 9.8 MeV/u C-ions (Figure 2) and >2 Gy X-rays. Thus, the damage induced by IR in first division cells clearly depends on the radiation quality and dose. Apoptosis was assessed 48 h after radiation exposure by investigation of morphological criteria of the cell nuclei. A slightly yet insignificantly increased fraction of apoptotic cells was observed in the irradiated samples independent of dose or radiation quality (Figure 3).

### Radiation-Induced Damage Is Transient Rather Than Persistent in Cells Cultured up to 46 Days Following Exposure

For investigation of putative late effects of IR, we cultured both exposed cells and sham-irradiated cells up to 46 days corresponding to 22 population doublings post-irradiation (Figure S1 in Supplementary Material). Generally, radiation exposure did not severely alter the population growth compared to the control. Only in one case, i.e., after exposure to 1.5 Gy X-rays, a slightly lower CPD was found toward the end of the culture time.

**Figure 4:**
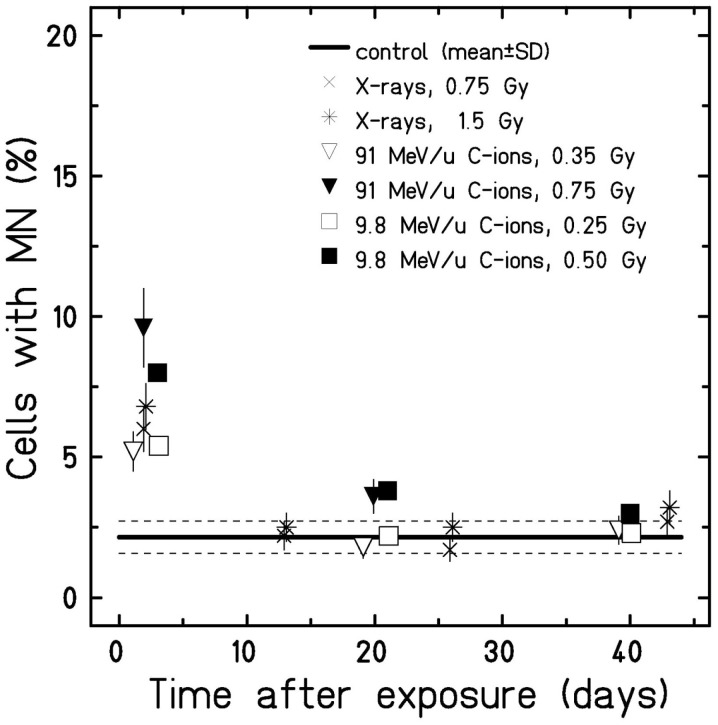
**Micronuclei formation in cells after extended culture time**. Cells were fixed at several time points after exposure (w/o cytochalasin-B), and all cells harboring micronuclei were scored. The error was calculated according to Poisson statistics, and a Fisher’s exact test was performed (*n* = 1). Only 2 days after exposure, micronuclei formation was found significantly higher compared to the control (mean ± SD). Note that for better visualization, the samples 2 days after exposure to the different radiation types were plotted separated from each other despite stemming from the same time point.

Next, we determined the amount of cells harboring micronuclei after an extended culture time (Figure 4). To allow for a better comparison, we plotted the mean value (±SD) of all controls over time instead of single data points. As shown in Figure 4, in all irradiated samples, the fraction of HUVEC containing micronuclei was significantly increased 2 days after exposure. For C-ions, the increase was dose dependent. Generally, at the following time points, only small differences between irradiated and sham-irradiated control cultures were found. Yet, exposure to the high doses (0.5 and 0.75 Gy) low and high energy C-ions resulted in an increased fraction of cells containing micronuclei when comparing to the respective controls (not displayed) 21 and 20 days post-irradiation, respectively. These increases are above the range of the mean value of pooled controls from all experiments and its upper SD, as indicated in the graph (Figure 4). Subsequent investigation time points did not reveal significant dose effects compared to the controls. Thus, analysis of the formation of micronuclei provided no evidence for a radiation-induced chromosomal damage in the progeny of irradiated cells.

Furthermore, we investigated radiation-induced apoptosis in the descendants of irradiated cells. The morphological analysis of the cell nuclei showed no differences in the fraction of apoptotic cells in irradiated samples compared to the respective controls (data not shown).

Additionally, the putative damage on the MMP was studied by applying the proton-sensitive dye JC-1. In control cultures (*n* = 4), the proportion of cells with an intact MMP (mainly red-fluorescing cells) amounted to 79 ± 8.4% (mean ± SD) over the whole time interval investigated (data not shown). After exposure to X-rays or C-ions, we found a slight decrease in cells with an intact MMP between 3 and 8 days post-irradiation, partly falling below the value of the lower SD (i.e., about 71%) down to 61% (for 1.5 Gy X-rays and 0.35 Gy C-ions 91 MeV/u, data not shown). To elucidate whether higher doses are required to profoundly impair mitochondrial function in HUVEC cultures within this period of time, we exposed cells to 1.5, 4, and 10 Gy X-rays and analyzed the MMP daily until 10 days after exposure (Figure S2 in Supplementary Material). We found that 2 days after exposure for all three doses applied, the amount of cells exhibiting mainly red fluorescence was lower compared to the respective control and the lower SD of all controls. Three days after irradiation with 1.5 Gy X-rays, the fraction of cells containing mitochondria with mostly intact MMP rose and reached the control level by day 5. For cells exposed to 4 Gy X-rays, recovery started at day 6 and the control value was reached by day 7, whereas the exposure to 10 Gy resulted in a persistently decreased level of cells with an intact MMP over the period investigated. Hence, the dose dependence was expressed rather in the recovery time than in the fraction of cells with intact MMP.

Furthermore, the expression of SA-β-gal was investigated in the progeny of irradiated and non-irradiated HUVEC to assess whether the radiation exposure induced premature senescence. Generally, the fraction of SA-β-gal positive cells raised with an increasing CPD. The proportion was comparable in irradiated samples and the respective controls. Only in one sample, i.e., 6 days after exposure to 0.5 Gy C-ions 9.8 MeV/u, an increased fraction of SA-β-gal positive cells was found and thus may be considered false positive (Figure S3 in Supplementary Material). Altogether, these data indicate that within the dose range investigated neither X-ray nor C-ion exposure induces a premature cellular senescence of HUVEC cultures.

### Analysis of Chromosomal Aberrations by the mFISH Technique Revealed Specific Alterations in the Progeny of Non-Irradiated and Irradiated HUVEC Cultures

**Figure 5:**
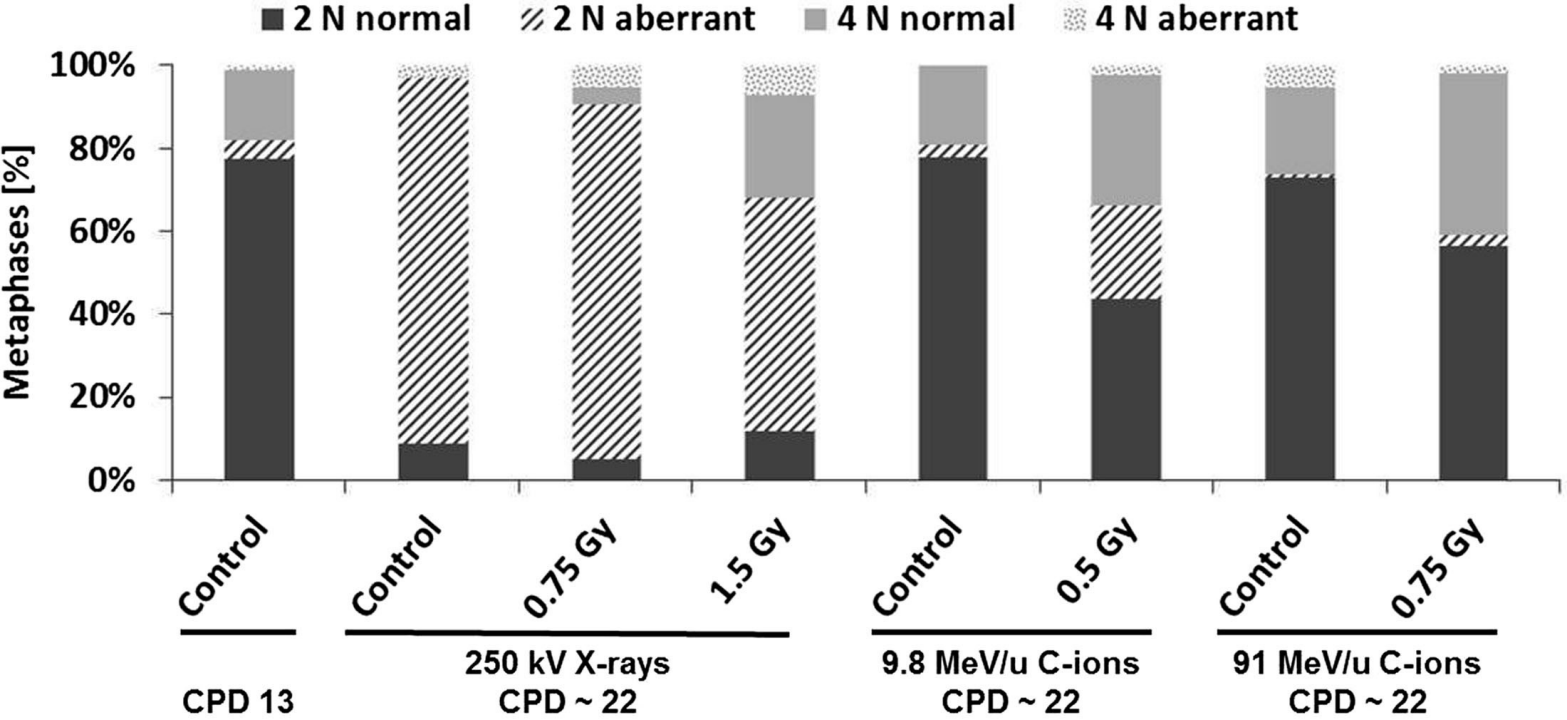
**Analysis of structural chromosome aberrations in HUVEC cultures (non-irradiated and irradiated) by means of the mFISH technique**. The fractions of normal and aberrant diploid/hypodiploid cells (referred to as ~2N) and tetraploid/hypotetraploid cells (referred to as ~4N) are given (*n* = 1). The terms hypodiploidy or tetradiploidy indicate the loss of one or two chromosomes. Cells were analyzed in controls at a CPD level of 13 ± 2 and about 9 population doublings after exposure (CPD ~22).

To verify the observation that the progeny of non-irradiated and irradiated cells do not express an elevated level of cytogenetic damage (Figure 4), we measured chromosome aberrations in all cultures about 9 doublings post-irradiation corresponding to a CPD level of ~22. Additionally, the baseline level of aberrations was determined (CPD level ~13). The analyses were performed by means of the high-resolution mFISH technique. As shown in Figure 5, in non-irradiated HUVEC cultures at CPD ~13 most cells had a normal (2N) karyotype, occasionally the loss of one chromosome was observed. Overall, about 80% of the cells were diploid or hypodiploid. Notably, also tetraploid cells (4N) and a few cells with a hypotetraploid karyotype were registered (in total 20% of the population). Structural aberrations (mainly breaks and translocations) were detected in ~5% of cells analyzed. With increasing CPD, only small changes occurred in two control cultures (C-ion studies), but chromosome 13 appeared to be non-randomly involved. Either it was truncated or one copy was lost. In one control culture (X-ray study), the proportion of cells with a ~2N karyotype was much higher at CPD ~22, i.e., amounted to 97%. Notably, also the number of cells with structural aberrations was highly elevated, i.e., 88/97 ~2N cells and 3/3 ~4N cells were aberrant. In all affected cells, the same aberration, a large truncation of the q-arm of chromosome 13, was observed indicating a clonal origin.

**Figure 6:**
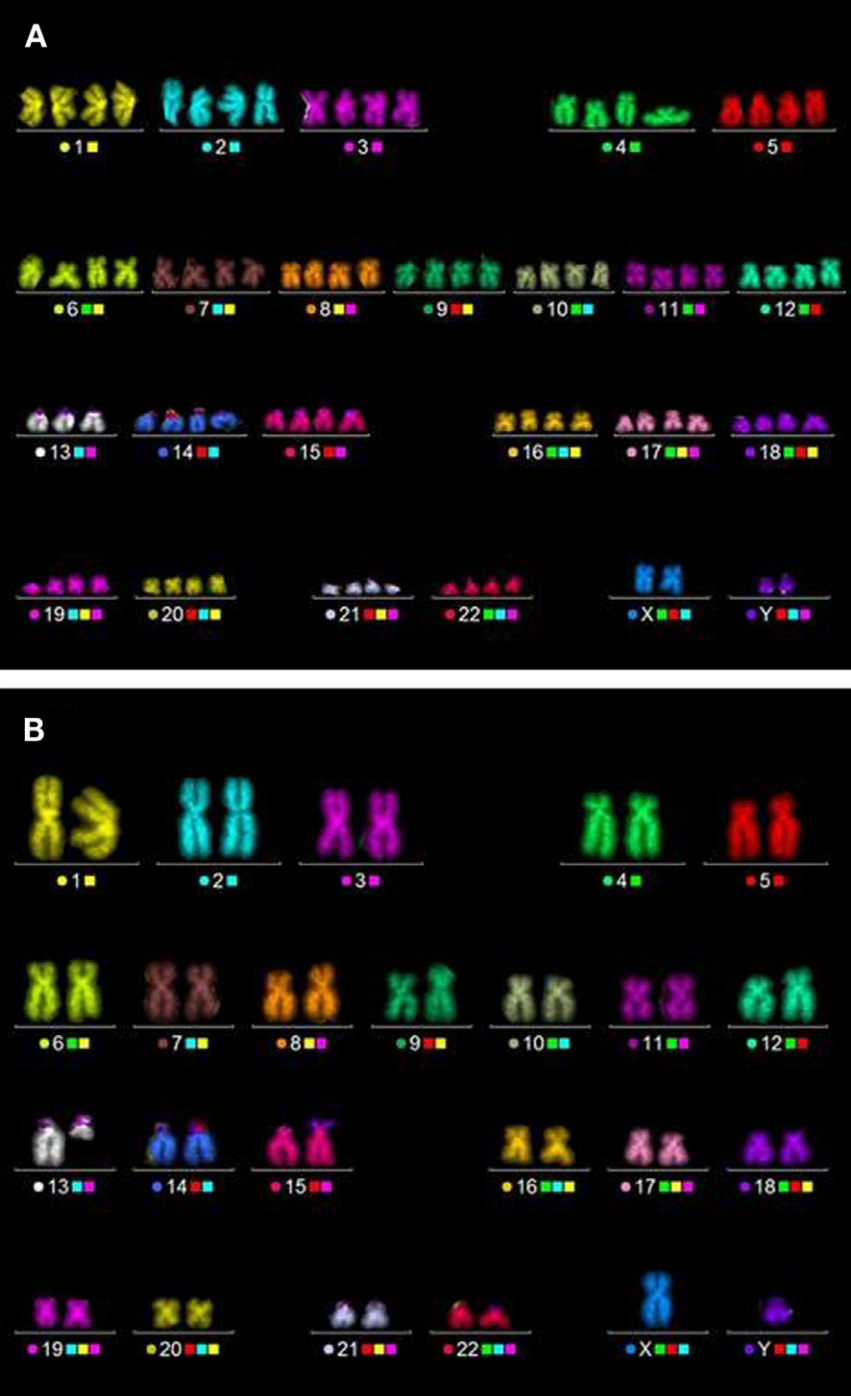
**Typical aberrations detected in HUVEC by means of the mFISH technique**. **(A)** Hypotetraploid cell, one copy of chromosome 13 is lost. **(B)** Diploid cell, one chromosome is truncated (here: non-irradiated cells, CPD ~22).

Chromosome analysis in cells at CPD ~22 (Figure 5) consistently showed that the fraction of ~4N cells was generally higher in the progeny of irradiated cells than in the respective controls culture. Structural aberrations were found in all cultures and were generally translocations (sporadic or clonal) or truncated chromosomes. As observed in the control cultures chromosome 13 was non-randomly involved in aberrations. Likewise, the loss of one or two chromosomes was registered. Again, chromosome 13 was over-represented (Figure 6). A summary of the data is shown in Table S2 in Supplementary Material.

Altogether, these data show that in HUVEC chromosome 13 is inherently unstable. Frequently, cells with a lost or truncated chromosome were observed. Based on the number of cells affected (i.e., the clone sizes), the loss or the deletion of a large part of the q-arm of chromosome 13 results in a survival advantage. As these changes are clonal they remain undetected by micronucleus analysis.

## Discussion

Epidemiological data demonstrate an increased risk of CVD when the heart and its adjacent blood vessels are exposed to relatively high doses of low-LET radiation as a consequence of radiotherapy, e.g., breast cancer or Hodgkin lymphoma (1, 2, 30). An increased risk of CVD at low or moderate doses of IR is indicated by epidemiological data stemming mainly from atomic bomb survivors or occupationally exposed groups (7, 31). However, the mechanisms leading to CVD after exposure to IR remain to be elucidated. The available data point at damage to the endothelial cells as the initial event in pathogenesis (31). Hence, we chose HUVEC as a model system. This system bears two advantages. First, the umbilical cord provides a cost-effective source of endothelial cells. Second, in several studies, the effect of low LET on HUVEC has already been examined [e.g., Ref. (32, 33)]. Since data on high-LET C-ions are scarce but of great interest owing to the increased use of C-ions in modern radiotherapy (15–17), we analyzed the response of HUVEC after exposure to C-ions with energies relevant for radiotherapy (see Table S1 in Supplementary Material).

### Radiation Induces a Dose- and LET-Dependent Damage in HUVEC 24 h Following Exposure

In HUVEC, radiation-induced damage in terms of clonogenic survival was found to depend on both dose and LET (Figure 1). For 91 MeV/u C-ions, an RBE value of 1.5 was obtained, whereas 9.8 MeV/u C-ions resulted in an RBE of 2.4. This is in line with the data reported for other cell lines [e.g., Ref. (34, 35)]. Cell survival of HUVEC after exposure to low-LET radiation was already measured by others (36, 37), but the radiosensitivity of cells used in the present study was much higher. For example, in the present study, a surviving fraction of 10% was reached after exposure to 2 Gy X-rays (Figure 1), whereas 4 and 5 Gy were needed for the same effect in the studies of Manti et al. and Hei et al., respectively. Furthermore, the survival data published by both authors show a shoulder, typically observed after exposure to low-LET photons. By contrast, our X-ray data display no shoulder. Lack of a shoulder points to a higher radiosensitivity and might be caused, for example, by a reduced DNA repair capacity as reported for Ku80-deficient cell lines [e.g., Ref. (34)]. Since HUVEC originate from apparently healthy donors, it is unlikely that the observed difference in the shape of the survival curves is attributable to compromised DNA repair. Yet, one possible explanation is a difference in the cell culture condition. In the present study, a specialized medium for primary endothelial cells was used with 2% serum, while Hei et al. cultured HUVEC in medium with 20% serum. Manti et al. studied also the response of HUVEC after exposure to C-ions with different LET values (13 and 100 keV/μm) and reported a clear dose and LET dependence as found in our study, too. For C-ions with a high LET (100 keV/μm), Manti et al. did not find a shoulder either.

As observed for cell survival, C-ions were more effective than X-rays with respect to the formation of micronuclei in binucleated cells (Figure 2). Analogously, 9.8 MeV/u C-ions were more effective than 91 MeV/u C-ions due to the higher LET. The fraction generally increased with dose, however, after X-ray irradiation, a saturation was found for doses >2 Gy (data not shown). The available data indicate that for 9.8 MeV/u C-ions, the saturation occurred at a much lower dose (>0.5 Gy). Yet, for firm conclusions, measurements have to be performed over a wider range of doses. A dose-dependent increase in the rate of micronuclei in various rat, bovine, or human endothelial cell cultures (38, 39), and a saturation effect for doses around 2 Gy X-rays (38) has been reported by others and is in line with our findings (Figure 2). Since we screened for micronuclei in binucleated cells, the saturation may be correlated with a hampered cell division capacity for higher doses. Furthermore, cytochalasin-B is cytotoxic, thus an increased rate of apoptosis may compete with the formation of binucleated cells, additionally leading to an underestimation of the damage induced by IR.

In contrast to the damage induced as reduced clonogenic survival or micronuclei formation, apoptosis was found to be only slightly higher after exposure, independent from dose or applied radiation quality (Figure 3). A small increase in the fraction of apoptotic cells for HUVEC after exposure to low doses of X-rays is in line with literature (20).

Taken together, radiation does induce damage up to 24 h following exposure that depends both on dose and the LET value. Such damage to endothelial cells may be considered the initial event in the pathogenesis of CVD (31). However, CVD has a long latency period. Therefore, we investigated whether genetic damage persists in cultures and whether other cellular processes implicated in the pathogenesis of CVD were affected up to 46 days after exposure corresponding to 22 population doublings.

### Radiation-Induced Damage Does Not Persist

Genomic instability, disrupted mitochondrial function, and accelerated replicative cellular senescence are implicated in pathogenesis of arteriosclerosis (8, 11–14). To address this topic, we assessed micronuclei formation, occurrence of chromosomal aberrations, apoptosis, and changes in the MMP as well as the expression of SA-β-gal in HUVEC cultured up to 22 population doublings after exposure (Figure S1 in Supplementary Material). For follow-up investigations, we focused on low doses up to 0.75 and 1.5 Gy for C-ions and X-rays, respectively, comparable to each other by isosurvival levels.

A dose-dependent effect on the number of cells with micronuclei was still visible at 48 h following exposure (Figure 4). At the later time points, the fraction of cells with micronuclei was similar in irradiated and control cultures indicating that the genomic stability of the cells was not affected by IR. Genomic instability expressed as micronuclei formation is a known consequence of exposure to IR (40). Yet, to the best of our knowledge, no other data sets for endothelial cells cultured for a prolonged time post-irradiation are available for comparison.

Premature senescence is considered as a key cellular stress response resulting, e.g., from DNA damage (41–43). Likewise, data indicate that premature senescence may contribute to the pathogenesis of arteriosclerosis (44, 45). Along this line, we examined the activation of SA-β-gal, a marker of cellular senescence (46) in the progeny of irradiated HUVEC.

Generally, no radiation-induced alterations were found over the time course investigated when compared to controls (Figure S3 in Supplementary Material). Only in one sample (0.5 Gy of 9.8 MeV/u C-ions), an elevated number of SA-β-gal positive cells was found 6 days after exposure that did not persist. Published data for endothelial cells are in contrast to our results. For example, Grossi et al. (47) registered a higher number of SA-β-gal expressing HUVEC several passages after exposure to 1.75 Gy X-rays or 0.5 Gy C-ions (13 keV/μm), i.e., doses comparable to the one in the current study. An increased number of endothelial cells, including HUVEC expressing SA-β-gal, was also observed after exposure to higher doses (2–10 Gy) of X-rays (48–50). Reasons for this different response are still unknown. Yet, studies over an extended culture time consistently showed an increase in the number of SA-β-gal positive endothelial cells with cell age (47, 51, 52). Our data support this finding.

There is evidence that radiation-induced oxidative stress and hampered mitochondrial function (53) play a role in endothelial dysfunction and CVDs (31, 54, 55). For example, in heart tissue, an impairment of mitochondrial proteins related to oxidative phosphorylation (e.g., complex I and III) was demonstrated following exposure to ≤2 Gy X-rays (56). Therefore, we examined the MMP, a key parameter of mitochondrial function. The dye JC-1, whose red fluorescence is directly correlated with the integrity of the MMP (57), was applied (data not shown). We found a slight decrease in the amount of cells containing mainly mitochondria with an intact MMP shortly after exposure (i.e., up to 8 days) independent of the radiation type and dose. Yet, by applying higher doses of X-rays (1.5, 4, and 10 Gy), we recorded an impairment of mitochondrial function in HUVEC that increased with dose (Figure S2 in Supplementary Material). Likewise, a decrease of the MMP following staining with JC-1 or other fluorescent dyes after high doses of X-rays (≤10 Gy) was reported for other cell lines (58–60).

A stress-triggered decrease of the MMP may be related to the onset of apoptosis via cytochrome *c* release and subsequent signaling pathways (61). In this context, our findings collectively provide no evidence for a delayed radiation-induced apoptosis in HUVEC.

Interestingly, the number of structural and numerical chromosomal aberrations increased with culture time in the progeny of unirradiated and irradiated cells. Consistently, chromosome 13 was involved. While, to the best of our knowledge, truncation of chromosome 13 in HUVEC has not yet been described, its loss has already been reported by others (51, 62, 63) and was accompanied by a growth advantage, i.e., leading to clonal expansion. Our data show that not only the complete deletion of chromosome 13 but also a deletion of a large part of the q-arm of chromosome 13 confers a growth advantage to the affected cells. Noteworthy, the q-arm of chromosome 13 harbors the *Rb* gene, encoding for the Rb protein, a well-known tumor suppressor and regulator of the cell cycle (64) that may account for an enhanced replication. As the number of cells with cytogenetic changes increased with time in irradiated cultures and the respective controls in a similar way, it is reasonable to assume that these cytogenetic changes are a feature of aging HUVEC that is barely affected by IR within the dose range examined (0.5–1.5 Gy). Moreover, our data revealed considerable inter-experimental differences in the number and types of aberrations in HUVEC cultures at CPD level 22 (see Figure 4; Table S2 in Supplementary Material). Pronounced inter-experimental differences in the aberration yield were also reported for other cell types, e.g., human foreskin fibroblasts and skin fibroblasts, subcultured up to CPD level 50 (41). Reasons underlying this phenomenon remain to be elucidated.

Taken together, our data point to a radiosensitivity for HUVEC directly after exposure to radiation, i.e., mainly by cell killing and even low or moderate doses as used in this study result in a reduced cellular survival. This is important if endothelial cell damage is taken into account as the initial step in the pathogenesis of arteriosclerosis (31). It was hypothesized that endothelial cell damage may trigger pro-inflammatory signals, which finally results in the enhanced formation of arteriosclerotic lesions [e.g., Ref. (6, 31)]. Yet, the link between radiation-induced damage and pro-inflammatory signaling remains poorly understood and requires further investigation. Since we found C-ions LET dependently more effective (RBE of 1.5 and 2.4 for 91 and 9.8 MeV/u C-ions, respectively) than X-rays, our results demonstrate the need of further studies in order to better estimate a putative risk of high-LET radiation.

## Author Contributions

AH, RL, MD, and SR have substantially contributed to the conception and design of the work, as well as the acquisition, analysis, and interpretation of the data.

## Conflict of Interest Statement

The authors declare that the research was conducted in the absence of any commercial or financial relationships that could be construed as a potential conflict of interest.

## Abbreviations

CPD, cumulative population doublings; HUVEC, human umbilical vein endothelial cells; JC-1, 5,5′,6,6′-tetra-chloro-1,1′,3,3′-tetraethylbenzimidazolyl-carbocyanine iodide; LET, linear energy transfer; mFISH, multiplex fluorescence *in situ* hybridization; MMP, mitochondrial membrane potential; RBE, relative biological effectiveness; SA-β-gal, senescence-associated β-galactosidase.
