# Supplementary material for: The Influence of C-Ions and X-rays on Human Umbilical Vein Endothelial Cells
Source: Front Oncol. 2016 Jan 20;6:5. doi: 10.3389/fonc.2016.00005 (PMC4718996; doi:10.3389/fonc.2016.00005)
Supplement: Supplementary file 1 [file datasheet_1.docx]

Supplementary Material

**The influence of C-ions and X-rays on human umbilical vein endothelial cells**

**Alexander Helm^*^, Ryonfa Lee, Marco Durante and Sylvia Ritter**

*Corresponding author: a.helm@gsi.de


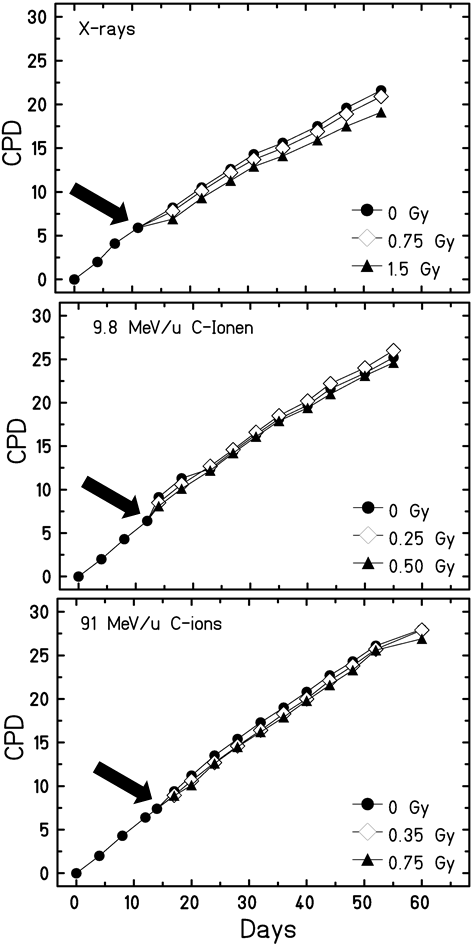


**Figure S1.** Cumulative population doublings (CPD). HUVEC were exposed to X-rays, 9.8 MeV/u or 91 MeV/u C-ions (n = 1) at a CPD about 6 (~ 11 days in culture, indicated by the black arrow) and subcultured up to a CPD of 28 (60 days in culture).


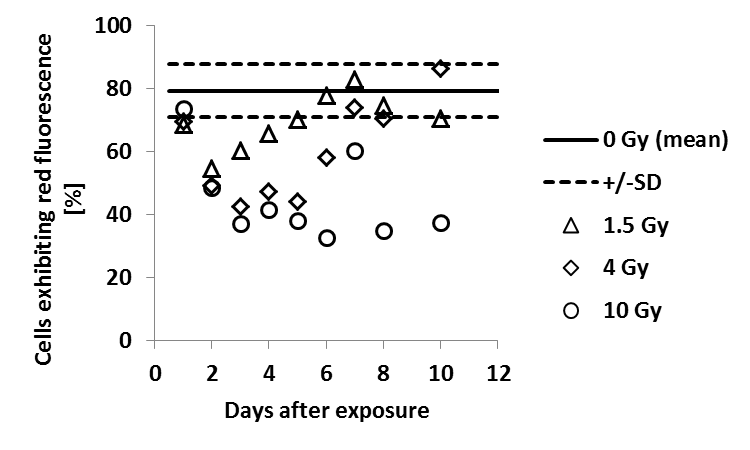


**Figure S2.** Mitochondrial membrane potential. Cells were stained with JC-1 dye prior to flow cytometer analysis at defined time points following radiation. The fraction of cells containing mitochondria with predominantly intact membrane potentials (indicated by red fluorescence) was determined. The mean±SD of four control cultures is plotted for comparison. Per sample, at least 1x10^4^ cells were analyzed (n = 1).


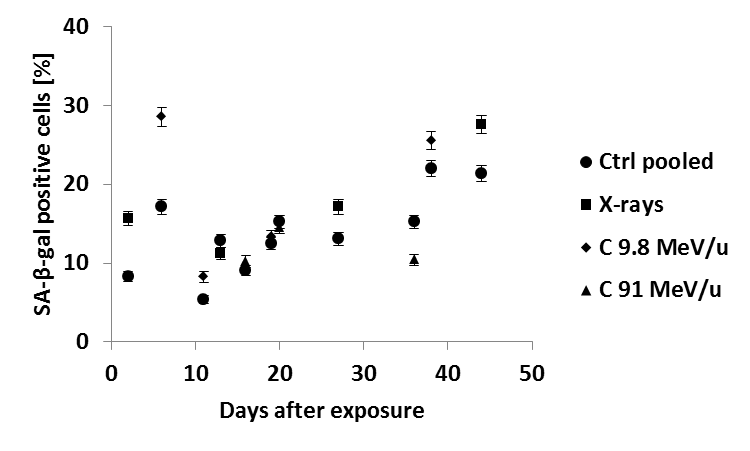


**Figure S3.** Expression of senescence associated β-galactosidase. Cells were fixed and stained at several time points after exposure. Cells positive for SA-β-gal, i.e. cells exhibiting a blue staining were scored. Controls of the three experiments are displayed as one set. The error was calculated according to Poisson statistics (n = 1).

**Table S1.** Supplementary information about applied high LET carbon ions.

| **Ion type** | **Energy on target (MeV/u)** | **LET (keV/µm)** | **Fluence (particles /cm^2^)** | **Dose (Gy)** | **Mean hits per nucleus** | **Number of cells hit at least once (%)** |
| --- | --- | --- | --- | --- | --- | --- |
| **Carbon** | 9,8 | 170 | 0.4 x 10^6^ | 0.1 | 0.3 | 27 |
|  |  |  | 1.0 x 10^6^ | 0.25 | 0.8 | 55 |
|  |  |  | 1.6 x 10^6^ | 0.4 | 1.3 | 72 |
|  |  |  | 2.0 x 10^6^ | 0.5 | 1.6 | 80 |
|  |  |  | 3.0 x 10^6^ | 0.75 | 2.4 | 91 |
|  |  |  | 4.0 x 10^6^ | 1 | 3.2 | 96 |
| **Carbon** | 91 | 28 | 0.2 x 10^7^ | 0.1 | 1.6 | 100 |
|  |  |  | 0.5 x 10^7^ | 0.25 | 4 | 100 |
|  |  |  | 0.7 x 10^7^ | 0.35 | 5.6 | 100 |
|  |  |  | 1.0 x 10^7^ | 0.5 | 8 | 100 |
|  |  |  | 1.6 x 10^7^ | 0.75 | 12.8 | 100 |
|  |  |  | 2.1 x 10^7^ | 1 | 16.8 | 100 |

**Table S2.** Supplementary information resulting from mFISH analysis.
